# Bifacial biological effects of ethanol: acetaldehyde production by oral *Streptococcus* species and the antibacterial effects of ethanol against these bacteria

**DOI:** 10.1080/20002297.2021.1937884

**Published:** 2021-06-09

**Authors:** Ryo Tagaino, Jumpei Washio, Haruki Otani, Keiichi Sasaki, Nobuhiro Takahashi

**Affiliations:** a Division of Oral Ecology and Biochemistry, Tohoku University Graduate School of Dentistry, Sendai, Japan; b Division of Advanced Prosthetic Dentistry, Tohoku University Graduate School of Dentistry, Sendai, Japan; c Division of Periodontology and Endodontology, Tohoku University Graduate School of Dentistry, Sendai, Japan

**Keywords:** Acetaldehyde, alcohol, antiseptics, bacteria, carcinogenicity, disinfectant, ethanol, oral cancer, *streptococcus* species

## Abstract

**Background:**Many previous studies have focused on the acetaldehyde produced from ethanol by oral bacteria as a risk factor for oral cancer. Most of these studies involved low ethanol concentrations (ca. 10 mM), but oral bacteria are exposed to a wide range of ethanol concentrations (100–10,000 mM) when alcoholic beverages are consumed. In contrast, ethanol is widely used at high concentrations (> 5,000 mM) as an antiseptic/disinfectant, suggesting that ethanol has bifacial biological effects; i.e. it acts as both a metabolic substrate for bacterial acetaldehyde production and an antimicrobial agent.

**Materials and methods:**We examined the acetaldehyde production from ethanol by oral streptococci and the effects of ethanol exposure on the growth and viability of these bacteria at a wide range of ethanol concentrations (10–10,000 mM).

**Results:**Acetaldehyde production was the highest at an ethanol concentration of 2,000 mM (2.1–48-fold higher than that seen at an ethanol concentration of 10 mM). Bacterial growth was inhibited by > 1,000 mM of ethanol, and the bacteria did not seem viable in the presence of > 5,000 mM of ethanol, although they still produced acetaldehyde.

**Conclusion:**Ethanol has bifacial biological effects, and the concentration ranges of these effects overlap.

## Introduction

Alcohol consumption, smoking, chronic mucosal irritation/trauma, and poor oral hygiene are considered risk factors for oral cancer [1–5]. Regarding the carcinogenicity of alcohol, the International Agency for Research on Cancer (IARC) classified alcoholic beverages into group 1 (carcinogenic to humans), since ethanol can be metabolized to acetaldehyde, a strong carcinogen [6–11]. The acetaldehyde present in the oral cavity is derived from alcoholic beverages themselves and their metabolism by the human body or oral bacteria [6]. Recently, acetaldehyde production by oral bacteria has been focused on as a risk factor for oral cancer since the oral mucosa is directly exposed to the acetaldehyde produced in oral biofilms [12–17].

It was reported that indigenous oral bacteria and yeasts, such as *Streptococcus, Neisseria*, and *Candida* species, produce acetaldehyde from ethanol [18–23]. Previously, we examined the acetaldehyde production by various oral streptococci at an oral ethanol concentration of 11 mM derived from food and drink and obtained the following findings: (1) the production of acetaldehyde was increased at a neutral to weak alkaline pH under aerobic conditions, (2) there were differences in acetaldehyde production among the streptococcal species, and (3) the production of acetaldehyde from ethanol involves the coupling of the ethanol oxidation reaction of alcohol dehydrogenase (ADH) with the oxidation reaction of NADH by NADH oxidase [24].

The concentration of ethanol in the oral cavity increases immediately after an alcoholic beverage is consumed and then decreases. It was reported after intake of an alcoholic beverage that the concentration of ethanol remaining in the oral cavity decreases gradually, as ethanol flows back into saliva from the blood for a few hours after it is taken into the body [25,26]. Most previous studies of acetaldehyde production by oral bacteria, including our study [24], used ethanol concentrations as low as 11–22 mM (approximately 0.05–0.1%), which corresponds to the ethanol concentrations seen in saliva a few hours after alcohol consumption [20,22,26,27]. However, during real-life alcohol consumption high concentrations of ethanol (5–10% = 1,000–2,000 mM) pass through the oral cavity, and hence, actual oral ethanol concentrations will often be higher than those used in the abovementioned studies. Therefore, it is necessary to assess the ability of oral bacteria to produce acetaldehyde at a range of ethanol concentrations.

On the other hand, ethanol is widely used as a disinfectant, as it is known to have bactericidal effects; i.e. it affects bacterial proliferation and viability [28–31]. However, it is unclear whether acetaldehyde is produced from ethanol by bacteria in the presence of the high concentrations of ethanol (≥5,000 mM) used in mouthwash and disinfectants [32–35].

In the present study, we investigated the production of acetaldehyde from ethanol by indigenous oral streptococci at a wide range of ethanol concentrations, from moderate ethanol concentrations similar to those seen after the consumption of alcohol beverages to high ethanol concentrations similar to those used in mouthwash or disinfectants. The effects of these concentrations of ethanol on the proliferation and survival of oral streptococci were also examined. Through these experiments, we attempted to clarify the details of acetaldehyde production from ethanol by oral streptococci and to show the bifacial biological effects of ethanol; i.e. that it has antibacterial effects but also acts as a substrate for bacterial acetaldehyde production.

## Materials and methods

### Bacterial strains and growth conditions

In this study, *Streptococcus mitis* (JCM 12971), *Streptococcus mutans* (NCTC 10449), *Streptococcus salivarius* (JCM 5707), *Streptococcus gordonii* (JCM 12995), and *Streptococcus sanguinis* (ATCC 10556) were used. The strains were grown and maintained on blood agar plates (CDC anaerobe 5% sheep blood agar, BD Japan, Japan) and were stored at 4°C in air. The strains were cultured in TYG medium containing 1.7% tryptone, 0.3% yeast extract, 0.5% NaCl, 50 mM potassium phosphate buffer (PPB) [pH 7.0], and 0.5% glucose at 37°C, and then 400 μL of the culture medium was transferred to new TYG medium (40 mL) and the strains were incubated further at 37°C.

### Assessment of bacterial population doubling times to estimate inhibitory effects of ethanol on bacterial growth

The bacterial stains were grown as described above until the logarithmic growth phase, and then each bacterial culture was divided into six cultures, and ethanol was added to each culture at a final ethanol concentration of 0 mM [0%], 10 mM [0.0465%], 100 mM [0.465%], 500 mM [2.33%], 1,000 mM [4.65%], or 2,000 mM [9.3%]. The cultures were continuously incubated at 37°C until the growth stopped. Bacterial growth was estimated based on the optical density (OD) of each culture at a wavelength of 660 nm, and population doubling times were calculated from the OD values. When the doubling time was ≥ 3.5 h, it was considered that no growth was occurring.

### Bacterial acetaldehyde production

In the logarithmic growth phase, the bacterial cells were harvested by centrifugation (10,000 rpm for 7 min. at 4°C) and washed three times with washing buffer (2 mM PPB [pH 7] containing 75 mM KCl, 5 mM MgCl_2_, and 75 mM NaCl), before being suspended in the same buffer. The bacterial cell concentrations of the suspensions were adjusted based on their OD at a wavelength of 660 nm (to an OD of 10).

The reaction mixture used for the assessment of bacterial acetaldehyde production (1,000 μL) contained 200 μL of the bacterial cell suspension, 0–486 μL of ethanol as a substrate (final concentration: 0 mM [0%], 10 mM [0.0465%], 100 mM [0.465%], 1,000 mM [4.65%], 2,000 mM [9.3%], 5,000 mM [23.3%], or 10,000 mM [46.5%]), and 50 μL of PPB (pH 7.0) in washing buffer. Each reaction mixture was kept in a tube with a silicone cap, and the tube was completely closed off. The reaction was started by adding ethanol, and the reaction mixture was incubated at 37°C for 30 min. The reaction was stopped by injecting 100 μL of 7 M phosphoric acid using a sterile needle (27 G × 3/4 Terumo injection needle, Terumo Corporation, Japan) and syringe (1 mL Terumo syringe for tuberculin, Terumo Corporation, Japan) through the silicone cap, and the mixture was shaken vigorously. The headspace gas of the tube was collected using a gas-tight syringe (10 mL Norm-Ject; Henke-Sass, Wolf; Tuttlingen, Germany). Then, the concentration of acetaldehyde was measured using a sensor gas chromatograph (SGEA-P2, FIS Inc., Japan). Since this gas chromatograph can precisely measure acetaldehyde concentrations ranging from 5 to 10,000 ppb, the samples were diluted with pure nitrogen gas, if necessary [24]. No acetaldehyde production was detected in control samples containing ethanol without bacterial cells.

### Bacterial viability after incubation with ethanol

The reaction mixture used to examine bacterial viability was prepared in the same way as that used to assess acetaldehyde production. After the reaction mixture had been incubated with ethanol at 37 °C for 5, 10, or 20 min. (5,000 mM or 10,000 mM of ethanol) or 30 min. (all ethanol concentrations), it was spread on TYG agar medium (containing 1.7% tryptone, 0.3% yeast extract, 0.5% NaCl, 50 mM PPB [pH 7.0], and 0.5% glucose) and incubated at 37 °C for three days. Then for the reaction mixtures with 5,000 mM and 10,000 mM of ethanol, the number of colony-forming units (CFU) that developed on each culture was determined.

### Statistical analysis

In the statistical analysis, the significance of differences among groups was analyzed using Tukey’s test, and p-values of < 0.05 were considered statistically significant (StatFlex Ver. 6).

## Results

### The effects of ethanol exposure on bacterial growth

**Figure 1.**
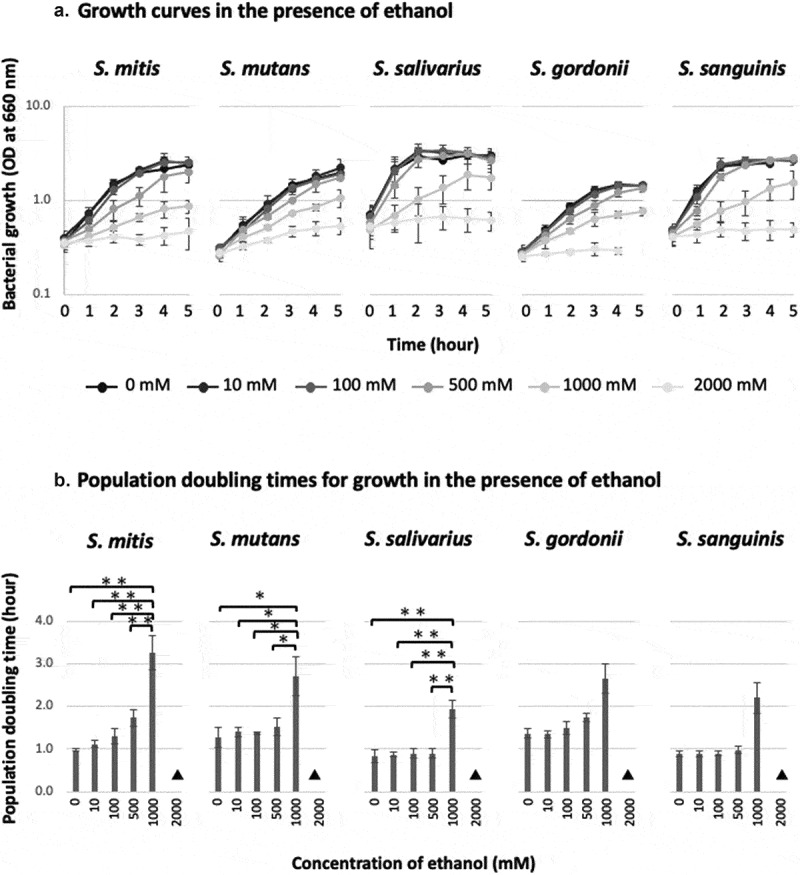
Population doubling times and growth curves of oral *Streptococcus* species in the presence of ethanol

In all five strains, ethanol inhibited the growth of the bacteria and increased the population doubling time. The bacteria failed to grow at ethanol concentrations of ≥ 2,000 mM. However, the inhibitory effect of ethanol on bacterial growth varied with the ethanol concentration and the bacterial strain. In *S. mutans, S. salivarius*, and *S. sanguinis*, the addition of 0–500 mM of ethanol did not inhibit bacterial growth, whereas the addition of 1,000 mM of ethanol caused the population doubling time to increase by more than two-fold; i.e. it markedly inhibited bacterial growth. On the other hand, in *S. mitis* and *S. gordonii*, the population doubling time gradually increased at ethanol concentrations of 0–500 mM and was markedly increased at an ethanol concentration of 1,000 mM. In *S. mitis, S. mutans*, and *S. salivarius*, significant differences in the population doubling time were seen between ethanol concentrations of 1,000 mM and 0–500 mM (Figure 1).

**A**. Growth curves of oral *Streptococcus* species in the presence of ethanol.Bars, standard deviation; n = 3.**B**. Doubling times of oral *Streptococcus* species in the presence of ethanol.Bars, standard deviation; ▲, A doubling time of > 3.5 hours was considered to be indicative of no growth. *p < 0.05, **p < 0.01; n = 3.

### Bacterial viability after incubation with ethanol

**Figure 2.**
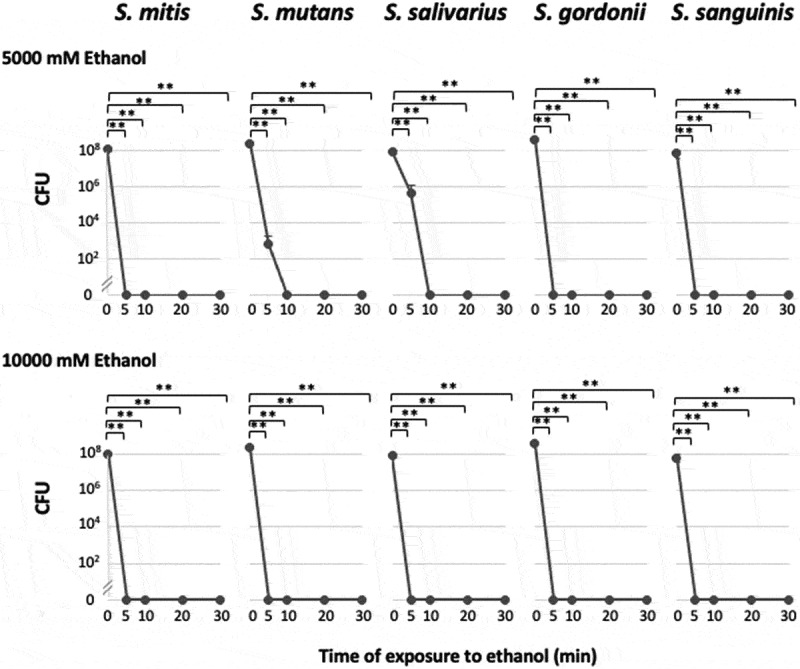
Bacterial viability (CFU) after exposure to 5,000 or 10,000 mM of ethanol

The viability of the bacteria after they had been incubated for 30 min. in the presence of ethanol (0, 10, 100, 1,000, or 2,000 mM) was confirmed by observing bacterial colonies on TYG agar medium. All the tested bacterial strains survived in the presence of 0–2,000 mM of ethanol. When the bacteria were incubated with 5,000 mM of ethanol for 5 min., only *S. mutans* and *S. salivarius* continued to grow; however, the numbers of CFU decreased significantly to < 1% of the control value. When the other strains were incubated with 5,000 or 10,000 mM of ethanol, they lost the ability to grow or exhibited significantly reduced survival (Figure 2).

Bars, standard deviation; **p < 0.01; n = 3.

### Acetaldehyde production from various concentrations of ethanol

**Figure 3.**
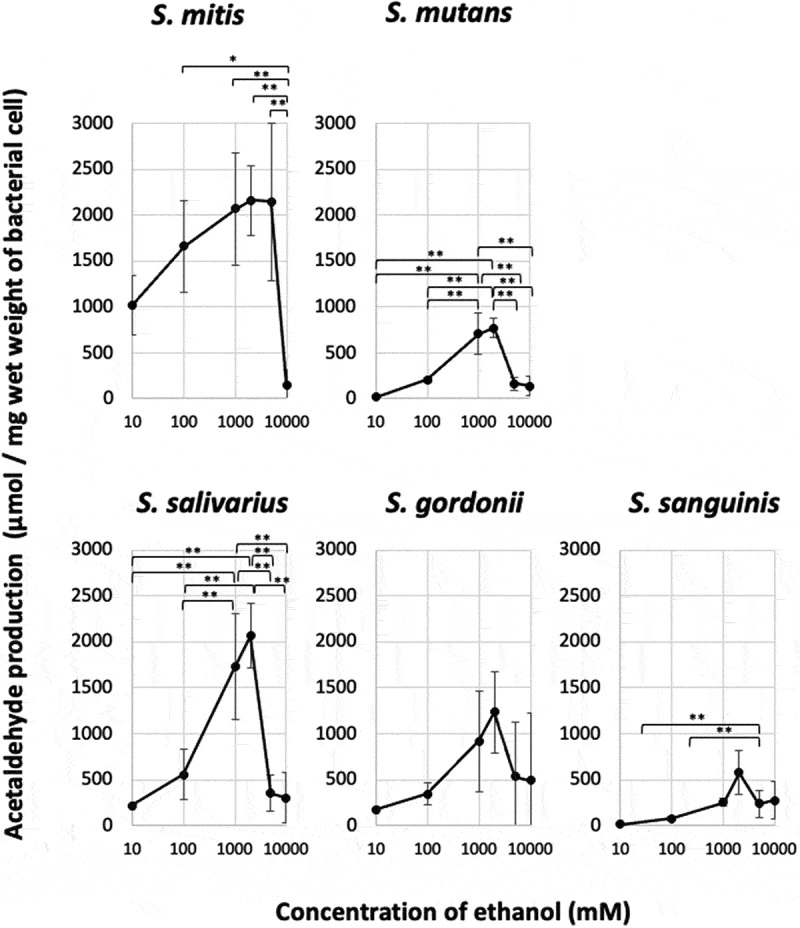
Acetaldehyde production from ethanol by oral *Streptococcus* species

In all strains, acetaldehyde production tended to increase with the ethanol concentration at ethanol concentrations of 0–2,000 mM and peaked at an ethanol concentration of 2,000 mM. Furthermore, when the ethanol concentration was increased to 5,000 or 10,000 mM, the acetaldehyde production tended to decrease (Figure 3). *S. mitis* and *S. salivarius* exhibited high maximum aldehyde production (V_max_) values, while *S. mutans, S. gordonii*, and *S. sanguinis* demonstrated low V_max_ values.

Bars, standard deviation; *p < 0.05, **p < 0.01; n = 3.

The ethanol concentration-dependent changes in acetaldehyde production were assessed by calculating the ethanol concentration that resulted in an acetaldehyde production value of half of the maximum acetaldehyde production value (C_1/2_). C_1/2_ varied among the strains. The obtained C_1/2_ values were 38.8 mM for *S. mitis*; 478.5–605.0 mM for *S. mutans, S. salivarius*, and *S. gordonii*; and 964.5 mM for *S. sanguinis* (Table 1). The C_1/2_ values of *S. mitis* and *S. sanguinis* were significantly different. *S. mitis* produced large amounts of acetaldehyde, even at low ethanol concentrations; and, unlike the other strains, *S. mitis* exhibited almost maximal levels of acetaldehyde production in the presence of 5,000 mM of ethanol (Figure 3).

**Table 1.** Maximum acetaldehyde production (V_max_) and the concentration of ethanol at which the production was half of the maximum acetaldehyde production (C_1/2_)

	*S.* *mitis*	*S.* *mutans*	*S.* *salivarius*	*S.* *gordonii*	*S.* *sanguinis*
The maximum acetaldehyde production (V_max_) (μmol/mg of wet weight of cells・min)	72.1 ± 12.7 ^a, b, c^	25.7 ± 3.5 ^a, d^	68.8 ± 11.8 ^d, e^	41.1 ± 14.8 ^b^	19.2 ± 8.1 ^c, e^
The concentration of ethanol at the maximum acetaldehyde production (mM)	2,000	2,000	2,000	2,000	2,000
The concentration of ethanol at which the production was half of the maximum acetaldehyde production (C_1/2_) (mM)	38.8 ± 15.4 ^f^	478.5 ± 70.0	488.2 ± 160.6	605.0 ± 316.4	964.5 ± 408.8 ^f^

a – e; significant differences between two bacterial species; e.g. ‘a’ shows that there is a significant differences between *S. mitis* and *S. mutans*.

a, c, d, e and f; p < 0.01, b; p < 0.05.

## Discussion

**Figure 4.**
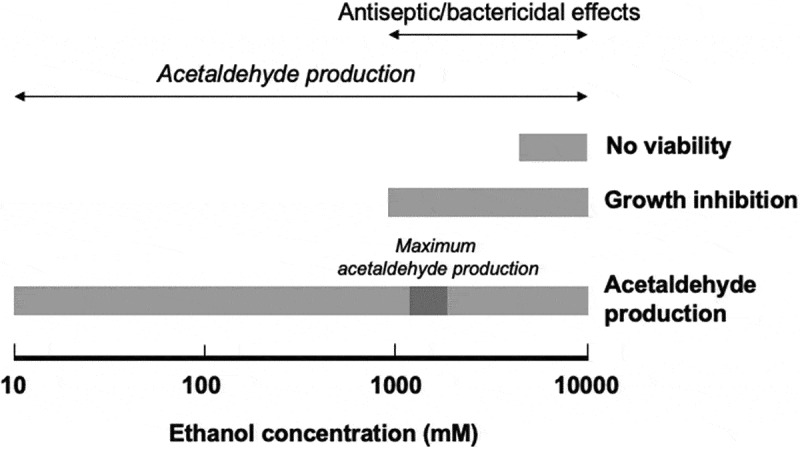
Bifacial biological effects of ethanol

The presence of 1,000 mM of ethanol inhibited the growth of oral streptococci (Figure 1), and incubating these bacteria with > 5,000 mM of ethanol for 10 min. seemed to abolish their viability (Figure 2), indicating that concentrated ethanol is bacteriostatic and bactericidal, as is well known [29,30,32] (Figure 4).

Ethanol functions as both a metabolic substrate for bacterial acetaldehyde production and an antibacterial agent. The maximum acetaldehyde production was seen at an ethanol concentration of about 2,000 mM. The growth of oral streptococcal species was inhibited by ≥ 1,000 mM of ethanol, and these species became non-viable at ethanol concentrations of 5,000 to 10,000 mM; however, acetaldehyde production by these species peaked at an ethanol concentration of 2,000 mM and was still seen at an ethanol concentration of 10,000 mM.

However, this study revealed that oral streptococci produce acetaldehyde at a wide range of ethanol concentrations, with maximal acetaldehyde production occurring at an ethanol concentration of 2,000 mM and some acetaldehyde production being seen at ethanol concentrations of 5,000–10,000 mM (Figure 3); i.e. at concentrations that have bacteriostatic (Figure 1) and bactericidal (Figure 2) effects on oral streptococci. These observations demonstrated that ethanol has antiseptic/bactericidal effects and acts as a metabolic substrate for acetaldehyde (a carcinogen) production by oral streptococci and that the concentration ranges for these effects overlap (Figure 4). These phenomena might have been due to the fact that ethanol increases the permeability of cell membranes. Flores et al. [36] showed that when 10% (2,150 mM) ethanol was added to the reaction mixture, intracellular β-galactosidase activity could be detected in intact yeast (*Kluyveromyces lactis*) cells, indicating that the ethanol had permeabilized the cell membrane, allowing enzyme substrates to cross the cell membrane and intracellular enzyme reactions to occur *in situ*. Similar phenomena may occur with oral streptococci [37].

The ethanol concentrations in alcoholic beverages vary from approximately 1,000 mM (4.65%) to 10,000 mM (46.5%), and it is known that immediately after the consumption of such beverages the concentration of ethanol in the oral cavity decreases to ≤ 100 mM (0.465%) due to dilution and rinsing with saliva, and it falls to approximately 10 mM (0.047%) after 2 or 3 hours [25]. Therefore, bacteriostatic and bactericidal effects cannot be expected at the ethanol concentrations produced by alcohol drinking, while it is considered that carcinogenic acetaldehyde is produced from ethanol by bacteria at these concentrations. Lachenmeier [38] suggested that the topical application of high concentrations of ethanol to the skin and oral cavity may induce acetaldehyde production by oral bacteria. Our findings support this possibility. Furthermore, Lachenmeier argued that the oral mucosa may be another source of acetaldehyde production because mucosal cells also exhibit ADH activity [38]. This may represent as a ‘trifacial function’ of ethanol in the oral cavity, and further studies of this are needed.

In this study, acetaldehyde production was measured at low to high concentrations of ethanol (0–10,000 mM), and the maximum acetaldehyde production (V_max_) and C_1/2_ were determined (Table 1). The strains used in this study were divided into those with low C_1/2_ values (*S. mitis*: < 40 mM), which produced relatively large amounts of acetaldehyde from low concentrations of ethanol; those with moderate C_1/2_ values (*S. salivarius, S. gordonii*, and *S. mutans*: 400–600 mM); and those with high C_1/2_ values (*S. sanguinis*: > 900 mM). A previous study found that in the presence of 11 mM (approximately 0.05%) of ethanol *S. mitis* produced the greatest amount of acetaldehyde, and *S. mutans* and *S. sanguinis* produced extremely small amounts of acetaldehyde; however, these findings may have been largely due to differences among the C_1/2_ values of each strain. In other words, while *S. mitis* can produce a large amount of acetaldehyde from a low concentration of ethanol because of its low C_1/2_ value, *S. mutans* and *S. sanguinis* can only produce acetaldehyde when the ethanol concentration is high because of their high C_1/2_ values. The V_max_ values of *S. mutans* and *S. sanguinis* were about 1/3 to 1/4 of that of *S. mitis* (Figure 3), and the contributions of these strains to acetaldehyde production at high ethanol concentrations are estimated to be high. Thus, it is suggested that at the low concentrations of ethanol (10 mM) seen a few hours after alcohol consumption, acetaldehyde production in the oral cavity is mainly due to *S. mitis*, with some contribution from *S. salivarius* and *S. gordonii*. On the other hand, in addition to *S. mitis, S. salivarius*, and *S. gordonii, S. mutans* and *S. sanguinis* seem to be involved in acetaldehyde production in the oral cavity right after an alcoholic drink with a moderate ethanol concentration (1,000–2,000 mM) is consumed. These results suggest that an ethanol environment is maintained in the oral cavity when alcohol is consumed over a long period of time, resulting in the production of more acetaldehyde by more bacterial species, thereby increasing the risk of cancer.

As mentioned above, ADH is involved in the production of acetaldehyde from ethanol [20,24,39,40]. Kurkivuori et al. [20] reported that the Km value for ADH in *S. mitis* is 758.3 mM, while the Km value for ADH in *S. salivarius* is 1.2 mM, which are not consistent with the C_1/2_ values calculated for these bacteria in the present study. Thus, the production of acetaldehyde by oral streptococci during ethanol metabolism may also be related to factors other than ADH activity, such as the efficiency of ethanol uptake into cells and NADH oxidase activity.

## Conclusion

We demonstrated that oral streptococcal species produced acetaldehyde from ethanol at a wide range of ethanol concentrations. *S. mitis* can produce large amounts of acetaldehyde at low ethanol concentrations, while *S. mutans* and *S. salivarius*, which produced relatively low amounts of acetaldehyde in a previous study, can produce acetaldehyde only when the ethanol concentration is high. Since ethanol concentrations of 1,000–2,000 mM, which can be achieved by drinking alcohol, had bacteriostatic effects, and ethanol concentrations of 5,000–10,000 mM, which can be found in disinfectants, had bactericidal effects, and these concentrations of ethanol resulted in significant amounts of acetaldehyde being produced, it was concluded that ethanol has bifacial biological effects, and that the concentration ranges of these effects overlap.
